# Differential Expression Profiles and Functional Analysis of Long Non-coding RNAs in Children With Dilated Cardiomyopathy

**DOI:** 10.3389/fped.2021.617298

**Published:** 2021-02-04

**Authors:** Dongxiao Cai, Bo Han, Wei Sun, Li Zhang, Jing Wang, Diandong Jiang, Hailin Jia

**Affiliations:** Department of Pediatric Cardiology, Shandong Provincial Hospital, Cheeloo College of Medicine, Shandong University, Jinan, China

**Keywords:** pediatric dilated cardiomyopathy, long non-coding RNAs, Microarray, gene expression profile, functional analysis, biomarkers

## Abstract

**Aim:** To evaluate the expression profile of long non-coding RNAs (lncRNAs) in different left ventricular function of dilated cardiomyopathy (DCM) in children and explore their possible functions.

**Methods:** The lncRNA microarray experiment was used to determine the differential expression profile of lncRNA in three children with DCM and three healthy volunteers. The functional analysis and the construction of the lncRNA-mRNA interaction network were carried out to study the biological functions. Quantitative real-time polymerase chain reaction (qRT-PCR) analysis was used to verify the microarray data.

**Results:** There were 369 up-regulated lncRNAs identified in the DCM patients (fold change >2, *P* < 0.05), and 505 down-regulated lncRNAs. Based on target gene prediction and co-expression network construction, 9 differentially expressed lncRNAs were selected for the PCR to verify the accuracy of the microarray data, of which 5 were up-regulated and 4 were down-regulated, and finally proved that 7 of them were consistent with the trend of microarray data results. Four of these lncRNAs had significant differences between the patients with poor cardiac function and patients with improved left ventricle function.

**Conclusion:** LncRNAs may play an important role in pediatric DCM and may provide a new perspective for the pathogenesis, diagnosis, and treatment of this disease.

## Introduction

Dilated cardiomyopathy (DCM) is the main cause of progressive refractory heart failure which necessitates heart failure and can lead to death (1). DCM is defined by the presence of a dilated left ventricle with systolic dysfunction in the absence of a hemodynamic cause which can lead to dilation and dysfunction (2). The annual incidence of DCM in children accounts for approximately 50% of pediatric cardiomyopathies (0.57/100,000) and is the most common type of pediatric cardiomyopathy (3, 4). The mortality rate of children with DCM is high, and most children with this condition die from heart failure (5). High-risk factors of children with DCM include decreased left ventricular function, left ventricular dilation, and left ventricular posterior wallthinning (6). However, the pathogenesis of DCM remains unclear yet. The existence of family aggregation in DCM suggests that genetics play a certain role in its pathogenesis. In recent years, literature has confirmed that familial DCM accounts for 20–50% of the total number of DCM cases (7). Further, an increasing amount of gene mutations have been found to be highly related to DCM, of which autosomal dominant inheritance accounts for 90% of all gene mutations including 16 genes, the 10 genes with the highest mutation frequency are listed in Table 1 (8, 9). But in general this may only account for a small part of the genetic cause. Another hypothesis supported by previous studies suggests that failure to eliminate pathogens or activation of chronic autoimmune mechanisms against cardiac antibodies after subacute and chronic viral myocarditis may finally foster the conversion to DCM (10). Therefore, further clarification of its etiology and pathogenesis is very important for clinical and scientific research.

**Table 1:** Genes associated with familial dilated cardiomyopathy.

**Gene**	**Protein**	**Mutation Frequency (%)**
TTN	Titin	20–25
LMNA	Lamin-A/C	5
MYH7	Myosin-7 (β-myosin heavy chain)	4
MYH6	Myosin-6 (α-myosin heavy chain)	4
TNNT2	Cardiac muscle troponin T	2
MYBPC3	Cardiac-type myosin-binding protein C	2
MYPN	Myopalladin	2
SCN5A	Sodium channel protein type 5 subunit α	2
RBM20	RNA-binding protein 20	2
PLN	Phospholamban	1

Long non-coding RNA (lncRNA) are usually defined as transcripts greater than 200 nucleotides in length of non-coding RNA (ncRNA). This ncRNA is a type of RNA that can be transcribed from the genome, but not translated into protein (11). LncRNA is comprised of tens of thousands or even hundreds of thousands of nucleotides, so it has an extremely complex secondary structure, and it can perform a variety of molecular functions such as signaling, decoy, guidance, and scaffolding by interaction with DNA, RNA, or proteins (12, 13). They can be located in the nucleus or in the cytoplasm and can be alternatively spliced or polyadenylated (12). It has been reported that lncRNA is indeed regulated in human cardiac diseases. Additionally, there is increasing evidence that lncRNAs play a vital role in vascular biology and cardiovascular diseases, such as myocardial infarction, cardiac hypertrophy and fibrosis, atherosclerosis, angiogenesis, and vascular remodeling (14). These lncRNAs can act as competing endogenous RNAs (ceRNAs) or natural microRNA sponges to bind miRNAs competitively for gene regulation (15) and can regulate co-expressed coding genes to affect gene expression by chromosomal looping (16) or by directly binding chromatin-modifying complexes (17) and transcription factors (18). In addition, the lncRNA Microarray Chip is an effective tool for high-throughput analysis of lncRNA expression and it has been used to study the expression profiles of lncRNA in many kinds of diseases (19). However, data related to DCM in children is sparse, and a few studies have focused on adult DCM and lncRNAs (20, 21).

In order to clarify whether lncRNA in peripheral leukocytes is associated with the occurrence of DCM in children, and if it correlates with progression or improvement of the disease, we used the Microarray Chip to analyze differential expression of lncRNAs and mRNAs in children with DCM and healthy volunteers.

## Materials and Methods

### Patients Selection and Sample Collection

Between March 2019 and December 2019, we selected 25 children with DCM (age 1 month to 17 years) and 25 healthy children at the Department of Pediatric Cardiology, Shandong Provincial Hospital Affiliated to Shandong University. All DCM cases were diagnosed clinically according to the Classification and Diagnosis of Cardiomyopathy in Children as set by the American Heart Association (2). The exclusion criteria were as follows: DCM after chemotherapy; tachycardia induced cardiomyopathy; secondary cardiomyopathy unless secondary to inflammation (alcoholic cardiomyopathy; DCM secondary to the immune system diseases and metabolic or endocrine disease); and DCM combined with other organ system diseases. The control group consisted of healthy children whose age and gender-matched those of DCM children.

We collected peripheral blood (3 mL) from the experimental and control groups, and also collected an additional 20 samples of peripheral blood samples from DCM patients with improved left ventricle function (LVEF > 45%) (22), including samples taken from the same patient during their recovery period. Blood was collected in anticoagulant EDTA tubes and store at 4°C as quickly as possible, generally within 2 h from collection, then white blood cells were separated and frozen into −80°C with TRIzol reagent. The baseline characteristics of the DCM group and the control group, and the clinical characteristics of the DCM group with poor and improved left ventricle function are presented respectively in Tables 2, 3, respectively. Three DCM samples (D1, D2, D3) and three normal samples (C1, C2, C3) were chosen for microarray analysis.

**Table 2:** Detailed information about the DCM patients and controls.

	**Median age (y), (Q1, Q3)**	**Sex**		**BP (mmHg)**		**Heart rate (rpm)**	**Laboratory examination**		**Echo parameters**	
		**Male,** ***n* (%)**	**Female,** ***n* (%)**	**SBP (mmHg)**	**DBP (mmHg)**		**Hs-TnT (pg/mL),** **(Q1, Q3)**	**BNP (pg/mL),** **(Q1, Q3)**	**LVEDD (mm)**	**LVEF (%)**
Healthy children (*n* = 25)	1.75 (0.92, 8.25)	12 (48)	13 (52)	96.08 ± 10.75	64.05 ± 7.16	110.24 ± 17.34	<3	<450	32.19 ± 5.12	64.08 ± 1.52
DCM children (*n* = 25)	1.42 (0.75, 6.75)	10 (40)	15 (60)	92.84 ± 12.1	57.44 ± 11.41	139.8 ± 32.47	42.48 (11.99, 82.36)	9,214 (3213, 16672)	47.7 ± 11.8	30.2 ± 8.6

*BP, blood pressure; SBP, systolic blood pressure; DBP, diastolic blood pressure; Hs-TnT, hypersensitive troponin T (normal range, 3–14 pg/mL); BNP, brain natriuretic peptide (normal range, 0–450 pg/mL); LVEDD, left ventricular end-diastolic dimension, tested by echocardiography; LVEF, left ventricular ejection fraction, tested by echocardiography (normal value >60%)*.

**Table 3:** Clinical characteristics about the DCM patients with poor and improved LV function.

	**DCM with poor LV function (*n* = 25)**	**DCM with improved LV function (*n* = 20)**
**BP (mmHg)**		
SBP (mmHg)	92.84 ± 12.1	95.45 ± 9.34
DBP (mmHg)	57.44 ± 11.41	63.1 ± 8.33
Heart rate (rpm)	139.8 ± 32.47	110.8 ± 14.37
**NYHA functional class**, ***n*** **(%)**		
I	–	13 (65)
II	–	5 (25)
III	3 (12)	2 (10)
IV	22 (88)	–
**Laboratory examination**		
Hs-TnT (pg/mL) (Q1, Q3)	42.48 (11.99, 82.36)	8.45 (4.98, 10.3)
BNP (pg/mL) (Q1, Q3)	9214 (3213, 16672)	121.25 (81.46, 329.75)
**Echo parameters**		
LVEDD (mm)	47.7 ± 11.8	38.09 ± 13.81
LVEF (%)	30.2 ± 8.6	54.75 ± 7.43

*NYHA, New York Heart Association*.

### Total RNA Extraction and Purification

Total RNA was isolated using SparkZol Reagent (Sparkjade, Qingdao, China) according to the manufacturer's instructions, and purified by using a RNeasy Mini Kit (Qiagen, GmBH, Germany). Then A NanoDrop ND-2000 spectrophotometer (NanoDrop, DE, USA) was used to check the quantity and purity of RNA. Qualified RNA is used in subsequent experiments such as microarray analysis.

### LncRNA Microarray Analysis

RNA samples of DCM and control groups were sent to Shanghai Sinomics Corporation (Shanghai, China) then used to generate biotinylated cRNA targets for the Sino Human ceRNA array V3.0. The slides were then used to hybridize with the biotinylated cRNA targets. After hybridization, slides were scanned on the microarray scanner. Data extraction was performed using feature extraction software 10.7 (Agilent technologies). The Quantile algorithm of the “limma” package in R was used to normalize the raw data. The data analysis was performed in accordance with the protocol specified by Agilent Technologies. A two-fold change cutoff was adopted.

### Analysis of RNA Sequencing Data

Genes with at least two-fold change in expression were selected for further analysis. To analyze their function, we predicted the *cis-* and *trans*-target genes of differentially expressed lncRNAs and analog gene ontology (GO) and Kyoto encyclopedia of genes and genomes (KEGG) pathway enrichments. The *cis*-target genes were selected based on the genes less than 10 kb away from lncRNA. The *trans*-target genes were predicted by complementary or similar sequences of lncRNA using the National Center for Biotechnology Information basic local alignment search tool (BLAST), the resulting complementary energy between the two sequences was calculated using RNAplex, and the sequences with *e* ≤ −30 were selected. The method of GO and KEGG pathway enrichment analyses used Fisher's exact test and the cluster Profiler data package from R were chosen; the selection criterion is the number of genes that fall on fold change ≥ 2, *P* < 0.05, and the term obtained in the drawing is based on the value of the enrichment factor sort in descending order of size. The resulting top 30 pathways were chosen.

In addition, we constructed the lncRNA-mRNA co-expression network using a k-core algorithm to determine which lncRNAs and mRNAs might play a pivotal role in the DCM and the relationship of them. We also constructed lncRNA-miRNA-mRNA network by predicting the miRNA molecules adsorbed by lncRNA.

### Quantitative Real Time-PCR

Quantitative real time-PCR (qRT-PCR) was performed by using LightCycler 480 (Roche, Shanghai, China) system to verify the accuracy of the data. The qRT-PCR was conducted according to the instructions of the SYBR® Green Premix Pro Taq HS qPCR Kit (AG11701, ACCURATE BIOTECHNOLOGY, HUNAN) specification. The primer sequences used in the qRT-PCR are illustrated in Table 4. The relative expression levels were presented using the 2^−ΔΔ*CT*^ method.

**Table 4:** Primers sequence for quantitative real time-PCR.

**Primer name**	**Primer sequence (5′-3′)**	**Length (bp)**
NONHSAT175499.1	F:TGTGCCTGATATAGTGCTTGGT	93
	R:TCTGTCTTCCTCACTAGCCTGT	
ENST00000560465	F:TGACTGGAAAACCCTCCCCA	128
	R:GCACTCCCACGTCTTATGCTC	
ENST00000596816	F:GCGAAGCTGTCATTAGCAAGG	138
	R:ACACAAACAGCATCCGTCTT	
NONHSAT215378.1	F:CGCAAACAGCAACCATAGCG	106
	R:AGCCTCACTTGGGAAGGAAGT	
NONHSAT252242.1	F:GGCAAGGCATTCTAACCCCAT	197
	R:AGCCCTCCAAGGTGTGTATGA	
NONHSAT137060.2	F:TTGTGAGACACTGGGGAGGT	250
	R:TGTCCTGCCTTTCCACATTCT	
ENST00000457996	F:ACCACGCATCCTGAGACAAAC	213
	R:TAGCCAAACCAACTGCCTCTG	
NONHSAT242978.1	F:CTGCAGCTGTTTCCAAGAGG	71
	R:TGGTAGGAGTCATGGAACCG	
ENST00000637940	F:ACCCCTCTCTCAACTGTCGG	91
	R:TCACTGCCAGAGGTCACCAA	

*F, forward; R, reverse*.

### Statistical Analysis

SPSS25.0 and GraphPad Prism 8.0 were used for data analysis. Continuous data in this article are expressed as mean ± standard deviation (SD), *n* (%) or median, and interquartile range. The difference between each group was compared by Student's *t*-test. *P* < 0.05 was considered as statistically significant.

## Results

### Expression Profiles of lncRNAs and mRNAs

**Figure 1:**
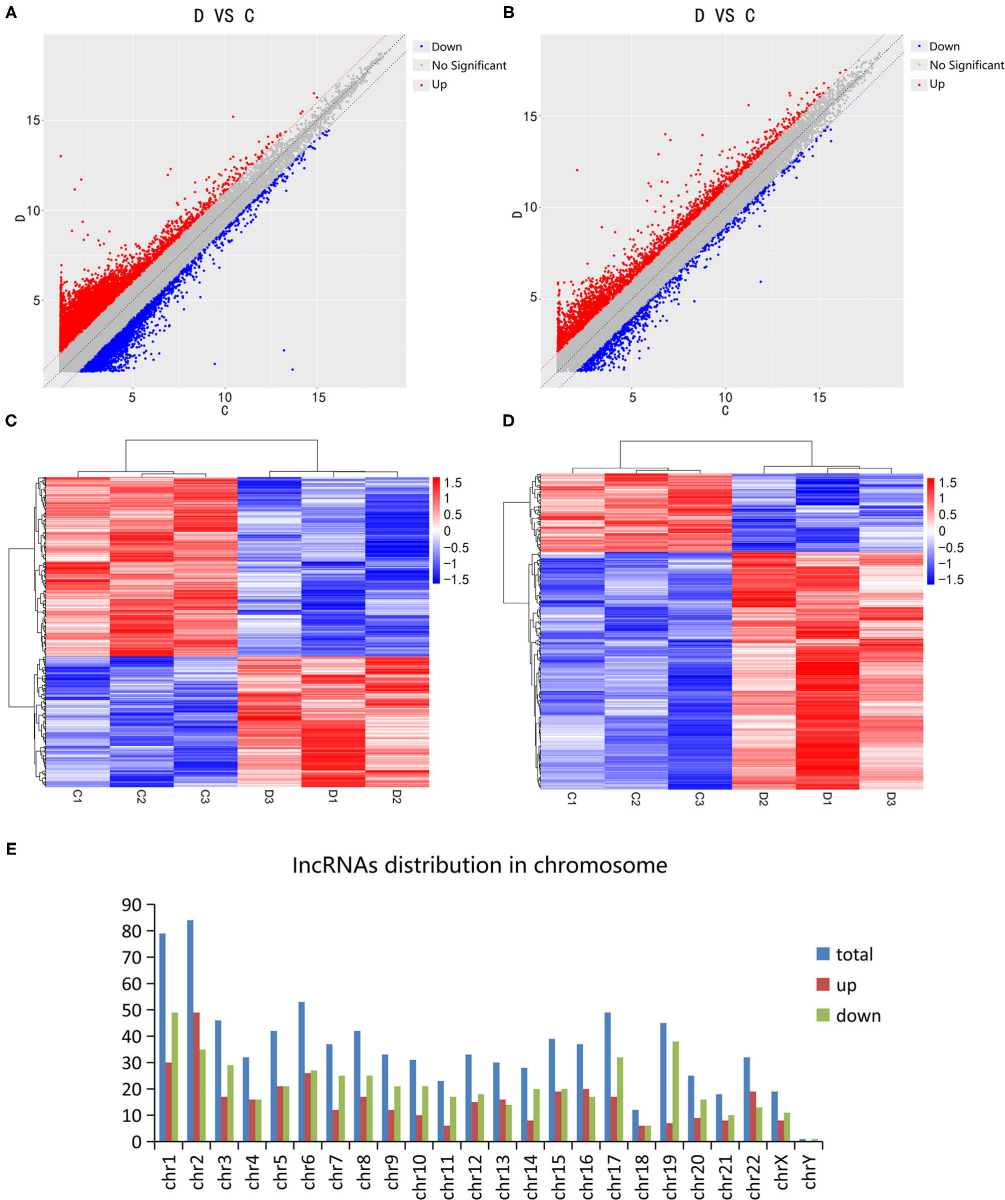
Expression levels of long non-coding RNAs (lncRNA) in dilated cardiomyopathy. Scatter plots were used to distinguish differentially expressed lncRNAs **(A)** and mRNAs **(B)**. Hierarchical cluster analysis of lncRNAs **(C)** and mRNAs **(D)** with altered expression (*P* < 0.05, fold change >2) between the two groups. Distribution of dysregulated lncRNAs in human chromosomes **(E)**. Red and blue represent upregulated and downregulated expression respectively. D indicates the experimental group; C indicates the control group.

The Microarray Analysis detected a total of 75,589 lncRNAs in 3 DCM samples and 3 control samples. The differential expression of lncRNA and mRNA in the DCM and control groups are shown as a scatter plot in Figures 1A,B. Additionally, the lncRNA and mRNA expression is also shown in a heat map using hierarchical clustering (Figures 1C,D). In summary, there are a total of 874 lncRNAs that are differentially expressed in patients with DCM as compared to the control group (fold change >2, *P* < 0.05), of which 369 are up-regulated and 505 are down-regulated. Figure 1E shows the distribution of these differentially expressed lncRNAs on human chromosomes. Moreover, there are 641 differentially expressed mRNAs between the DCM group and control group in all, which include 482 up-regulated and 159 down-regulated mRNAs.

### GO and KEGG Pathway Analysis

**Figure 2:**
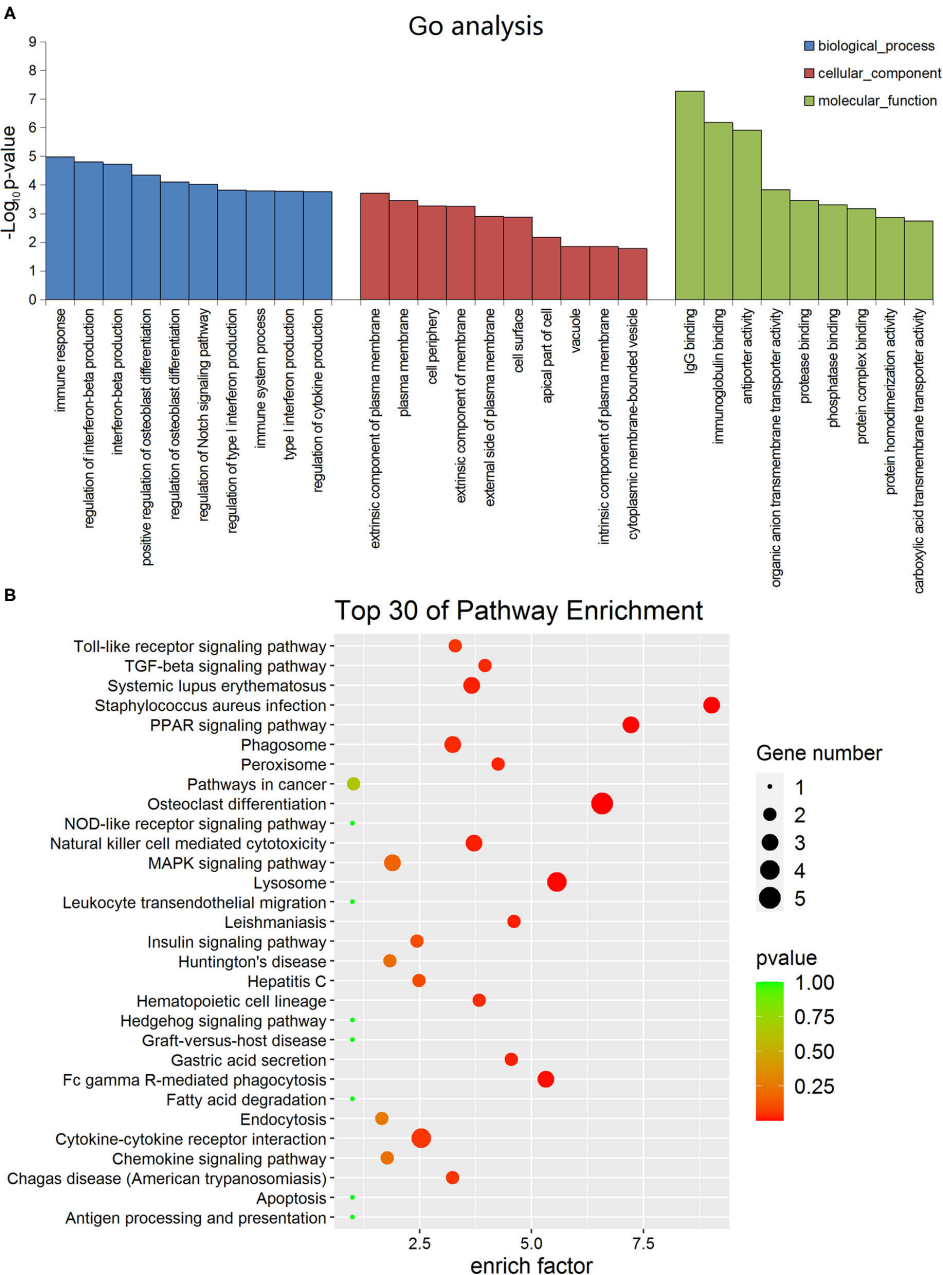
Gene ontology and Kyoto encyclopedia of genes and genomes pathway analyses of dysregulated long non-coding RNA (lncRNA)s in dilated cardiomyopathy. **(A)** The top 10 GO terms of the three different gene functions for lncRNAs. **(B)** The top 30 KEGG pathways for lncRNAs.

In order to determine the function of these lncRNAs more clearly, we performed GO analysis and KEGG pathway enrichment analysis. The GO enrichment analysis divides the function of genes into three parts: cellular component (CC), molecular function (MF) and biological process (BP). The top 10 of the three different gene functions of lncRNAs were shown in Figure 2A. Immune system and signal transduction are the most noteworthy pathways identified by KEGG classification. We selected the top 30 enriched KEGG pathways in the immune system and signaling pathways as shown in Figure 2B. We were more interested in “NF-kappa B signaling pathway,” “Toll-like receptor signaling pathway,” “PPAR signaling pathway,” “Apoptosis,” “Notch signaling pathway” and so on as they may be associated with DCM. Then we screened some differentially expressed genes in these pathways for future experiments.

### *Cis*- and *Trans*-lncRNA Target Gene Prediction

**Figure 3:**
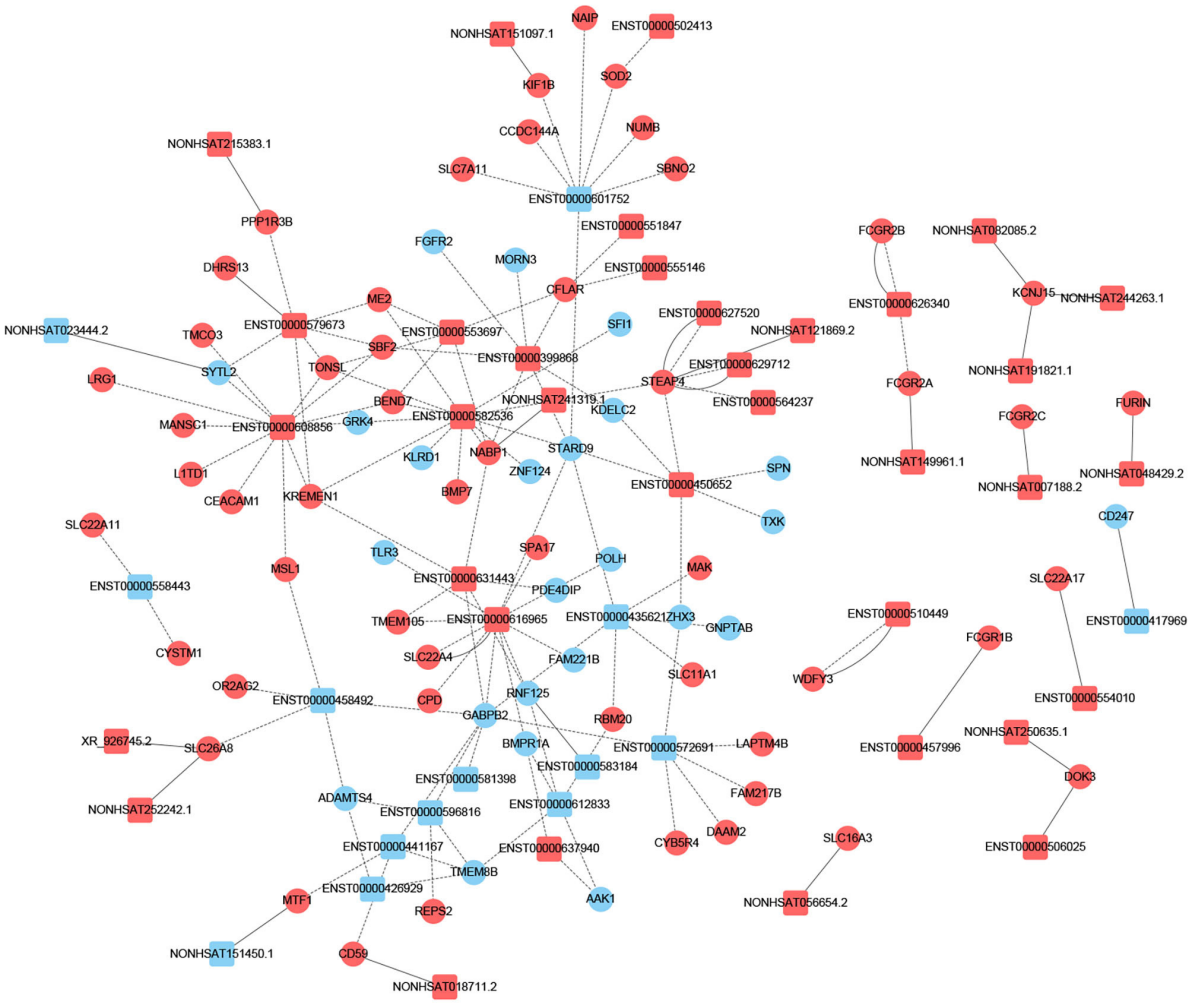
Prediction of *cis-*/*trans*-lncRNA targets in DCM-related pathways. The circles represent mRNAs, and the squares represent lncRNAs. Red means up-regulation, blue means down-regulation. The dotted line indicates a *trans*-lncRNA, and the solid line indicates a *cis*-lncRNA.

To determine if lncRNA could regulate gene expression, we predicted the target genes of lncRNA through different manners of action, including *cis* and *trans*. The principle of *cis*-predicting target genes is that lncRNA regulates the pattern of surrounding mRNA that is relatively close. On the contrary, the basic principle used when predicting *trans*-target genes is that the function of lncRNA has nothing to do with the position of the coding gene, but is related to the protein-coding gene that is co-expressed. We have performed a part of the lncRNA *cis*-/*trans*-target gene prediction in the pathway mentioned above as shown in Figure 3. In addition, according to the data, the *cis*-action manner of lncRNA accounts for the majority of the pathway.

### Construction of the Interaction Network Between lncRNA and mRNA

**Figure 4:**
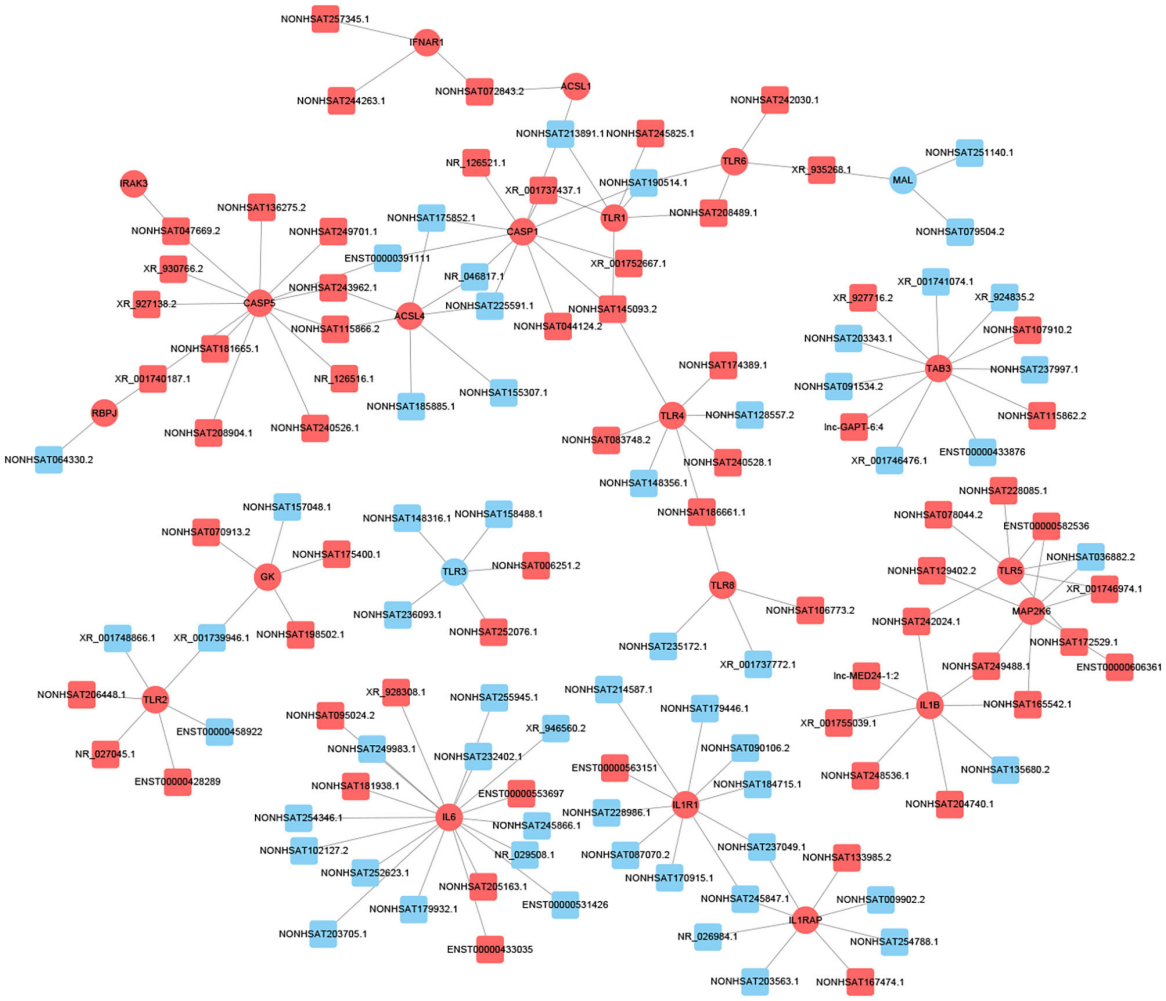
LncRNA-mRNA co-expression network analysis of the DCM-related pathways (Pearson's coefficient > 0.95). The circles represent mRNAs, and the squares represent lncRNAs. Red means up-regulation, blue means down-regulation.

**Figure 5:**
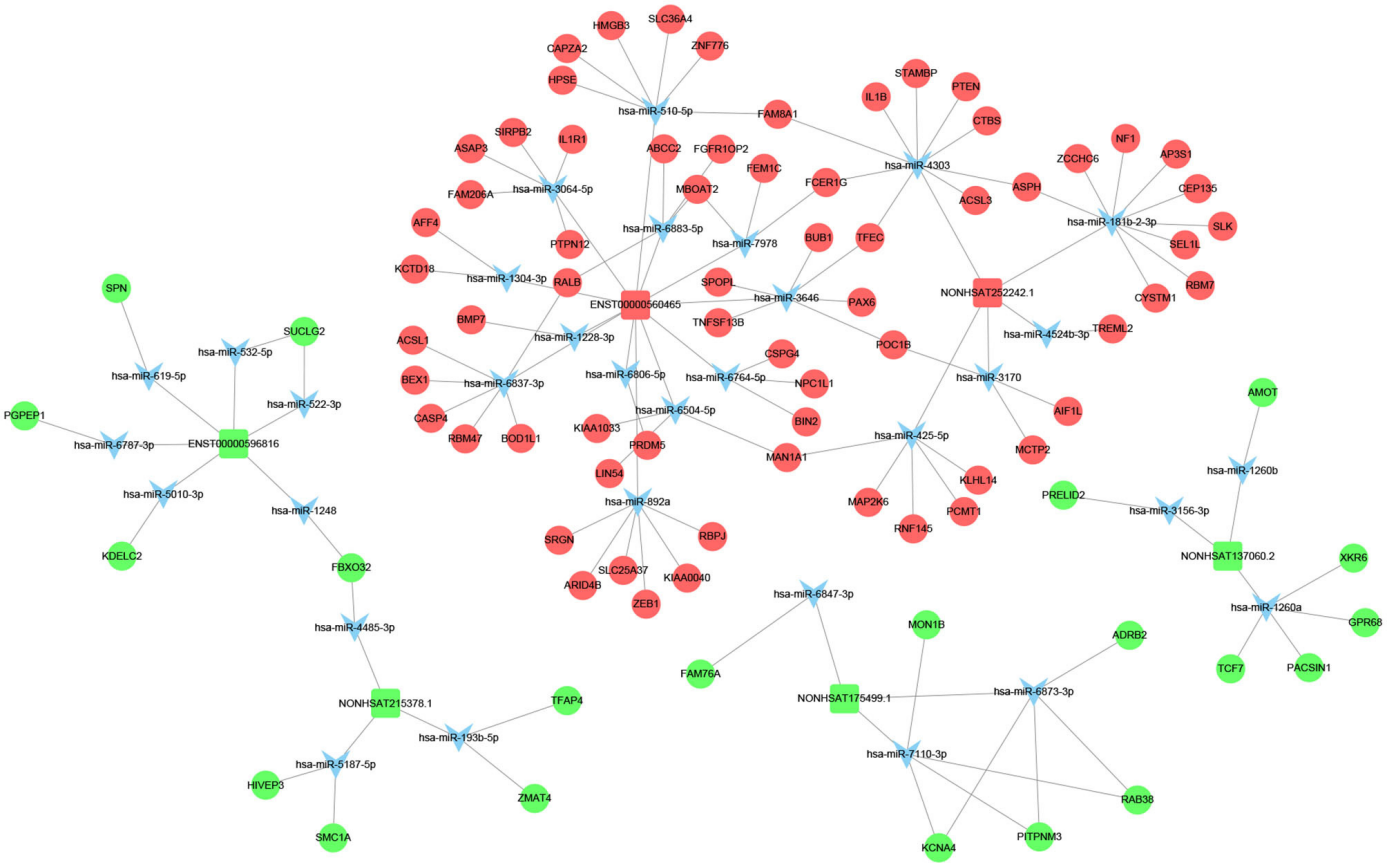
LncRNA–miRNA–mRNA interaction network. Inverted triangles represent the predicted miRNAs; rectangle nodes represent lncRNAs; circles represent mRNAs (red, upregulated; blue, downregulated).

Previous studies have shown that lncRNA can act as ceRNAs or natural miRNA sponges to bind miRNAs competitively to reduce the effects of miRNAs on target genes, ultimately achieve the goal that regulates target genes expression. On the other hand, by using the co-expression network, we can analyze the gene regulation ability and obtain the core regulatory genes obtained by the samples analyzed in this experiment. Using this information, we constructed the lncRNA-mRNA co-expression network and lncRNA-miRNA-mRNA network. We have identified 120 lncRNAs and 22 mRNAs altogether in the pathways mentioned above (Pearson's coefficient > 0.98). The lncRNA-mRNA network consisted of 142 net nodes and 149 connections of which each lncRNA was linked to 1–3 mRNAs, and each mRNA was linked to 1–18 lncRNAs (Figure 4). The five miRNAs with the highest binding potential for each lncRNA were predicted by using Cytoscape 3.5. The lncRNA-miRNA-mRNA network between the lncRNA related to the above pathways and the corresponding target mRNAs and target miRNAs was shown in Figure 5.

We screened out 9 lncRNAs based on the differentially expressed mRNAs and their different functions through the interaction network and the target gene prediction. The differentially expressed mRNAs involved in the above pathways include TLR3, PLIN4, PSEN1, BMP7, PPARG, etc. These 9 lncRNAs were selected for the next process.

### Validation of lncRNA Expression

**Figure 6:**
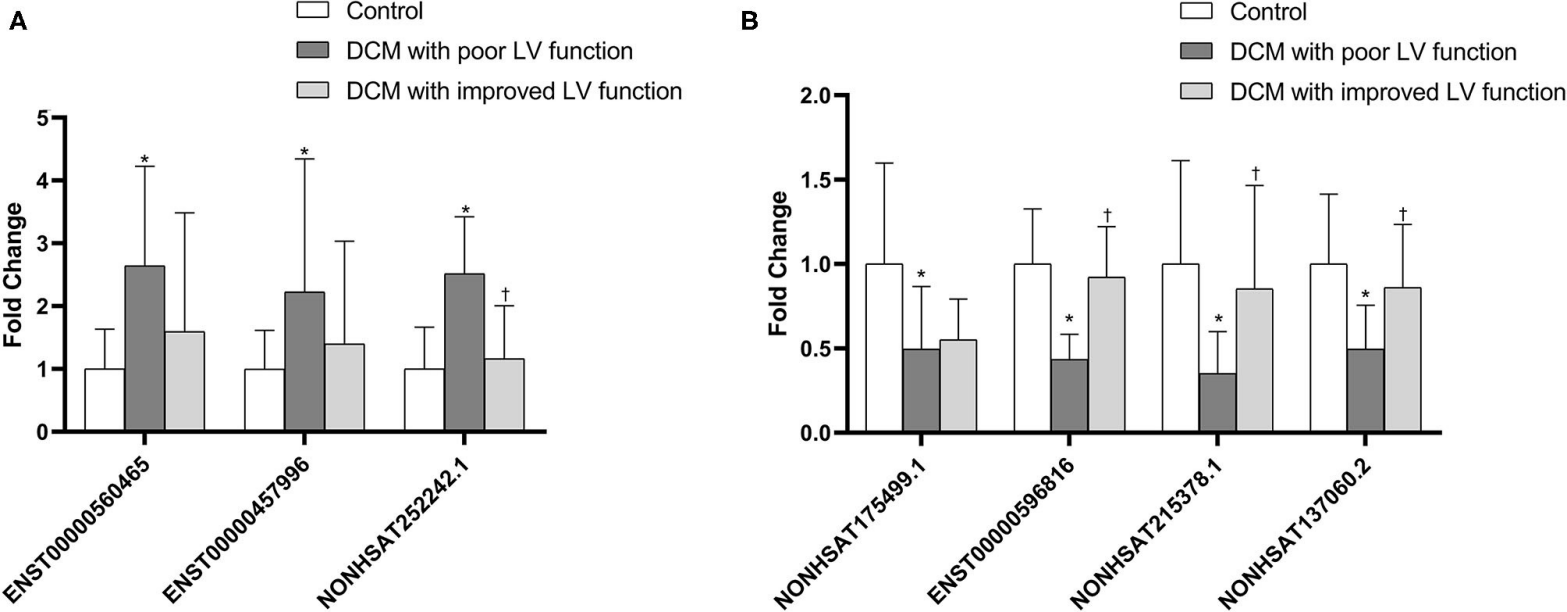
Comparison of the relative lncRNA expression levels between DCM patients and normal children. The expression of 3 upregulated lncRNAs **(A)** and 4 downregulated lncRNAs **(B)** were validated using the 2^−ΔΔ*CT*^ method. *P < 0.05 vs. control group. ^†^*P* < 0.05 vs. DCM with poor LV function.

As mentioned earlier, we screened out 9 lncRNAs, of which 5 were up-regulated and 4 were down-regulated. After qRT-PCR analysis, these 9 lncRNAs were all amplified individually. However, among them, compared with the control group, the result of NONHSAT242978.1 and ENST00000637940 in the DCM group were opposite to the trends shown by the microarray data. Among the other 7 lncRNAs, 3 up-regulated (ENST00000560465, NONHSAT252242.1, ENST00000457996) and 4 down-regulated (NONHSAT175499.1, ENST00000596816, NONHSAT215378.1, NONHSAT137060.2) were consistent with the trend of microarray data results and are statistically significant (*P* < 0.05) (Figure 6). We also verified if these lncRNAs are differentially expressed in DCM patients with poor cardiac function and DCM patients with improved left ventricular function. The results show that only NONHSAT252242.1, ENST00000596816, NONHSAT215378.1, NONHSAT137060.2 had significant differences (*P* < 0.05, fold change > 2) in the group with improved left ventricular function as compared to the other DCM group.

## Discussion

DCM is the most common type of pediatric cardiomyopathy in the clinic, which is a major cause of heart failure, and often progresses to become severe over time. End-stage heart failure often requires heart transplantation, for which the prognosis is poor. To date, the etiology and pathogenesis of most cases of DCM have not been clearly determined. Current studies generally believe that the cause may be related to genetics or immunity. The genetic mechanism may account for 20–50% of DCM. Most of the inheritance is autosomal dominant inheritance, and there are also recessive, X-linked and mitochondrial inheritance (maternal inheritance) (23). There are currently more than 100 genes related to DCM (24), of which TTN is the most common accounting for 11% of DCM cases (25). Another popular hypothesis about related mechanisms is that DCM develops as a result of autoimmune myocarditis (26). These hypotheses provide a platform for us to study the pathogenesis of DCM.

According to the position of lncRNA in the genome relative to the protein-coding gene, it can be divided into seven categories: sense strand, antisense strand, intron lncRNA, bidirectional lncRNA, intergenic lncRNA, enhancer RNA, and circular RNA (27). More and more lncRNAs have established functional relevance in cardiovascular diseases (28), and their high tissue-specific expression indicates that in theory they can be used as disease markers or therapeutic targets. At present, the expression profile and functional mechanism of lncRNA in children with DCM are still unclear.

In this study, we first explored the expression profiles of lncRNA and mRNA of white blood cells extracted from peripheral blood collected from 3 DCM children and 3 normal children. As mentioned above, a total of 874 lncRNAs have expression differences (*P* < 0.05, fold change > 2), of which 370 are up-regulated and 504 are down-regulated. Then, in order to prove the accuracy of the microarray analysis, we selected 9 differentially expressed lncRNAs based on the functions of the respective differentially expressed mRNAs, which were verified by qRT-PCR in 25 children with DCM and 25 healthy children. Seven of them were confirmed to show the same upward or downward trend of IncRNAs in the DCM and healthy groups, including three that were up-regulated (ENST00000560465, NONHSAT252242.1, ENST00000457996) and four that were down-regulated (NONHSAT175499.1, ENST00000596816, NONHSAT215378.1, NONHSAT137060.2).

In addition, we wanted to know whether the expression levels of these 7 lncRNA molecules were different between the participants in the acute phase of DCM and the participants with relative improvement of the left ventricle after treatment. Therefore, we collected 20 peripheral blood samples from DCM children with improved left ventricular function, and verified whether there was a difference in expression of each lncRNA between the acute phase group and the improved group by qRT-PCR. The experimental results prove that three of the lncRNA (NONHSAT252242.1, ENST00000596816, NONHSAT215378.1, NONHSAT137060.2) are significantly different in the above two groups (*P* < 0.05, multiples > 2). This finding indicates that the expression of these molecules may be related to the severity of DCM, and may be used as a biomarker to measure the degree of recovery of DCM in the future.

The GO and KEGG analyses were used to determine the potential biological functions of differentially expressed IncRNAs and mRNAs. Among them, the most significant pathways related to DCM include the PPAR signaling pathway, Toll-like receptor signaling pathway, and Apoptosis pathways, which are related to inflammation, immunity, apoptosis and fibrosis, and serve as the basis for the 9 lncRNAs that we initially selected. Then, we constructed the co-expression network of lncRNA-mRNA and the lncRNA-miRNA-mRNA network in the signal pathway mentioned above to obtain the relationship between IncRNAs and mRNAs. These findings may enable us to further understand the pathogenesis of DCM.

In summary, the above experimental results indicate that the mutual regulation between lncRNA and mRNA may be involved in the pathological process of DCM, and may be related to the improvement of left ventricular function which might suggest that these four molecules are more likely to become biomarkers related to DCM in children in the future.

It should be noted that there are some limitations in our study. Firstly, the sample size for microarray data analysis is small, and the false positive rate may be high. To account for this, we selected a larger number of samples for PCR validation, which helps to validate microarray data. Secondly, the part of the experiment here is mainly focused on bioinformatics analysis. The next step in future research experiments will be to select molecules according to the multiple to analyze for functional and mechanism verification.

## Data Availability Statement

The datasets presented in this study can be found in online repositories. The names of the repository/repositories and accession number(s) are: NCBI GEO SERIES (accession: GSE160986).

## Ethics Statement

The studies involving human participants were reviewed and approved by the Ethics Committee of Shandong Provincial Hospital Affiliated to Shandong University. Written informed consent to participate in this study was provided by the participants' legal guardian/next of kin.

## Author Contributions

BH and DC designed the study. DC and WS performed all experiments described. BH and DJ supervised the study. JW and HJ analyzed the data. DC wrote the manuscript. BH and LZ approved the final version of the manuscript. All authors contributed to the article and approved the submitted version.

## Conflict of Interest

The authors declare that the research was conducted in the absence of any commercial or financial relationships that could be construed as a potential conflict of interest.

## Supplementary Material

Supplementary Table 1 Differentially expressed lncRNAs in pediatric dilated cardiomyopathy.

Supplementary Table 2 mRNA profiles in pediatric dilated cardiomyopathy.

Supplementary Table 3 *Cis*-target genes of differentially expressed lncRNAs.

Supplementary Table 4 *Trans*-target genes of differentially expressed lncRNAs.

Supplementary Table 5 The prediction of co-expression network.

Supplementary Table 6 miRNA binding sites for circRNAs.
